# Supplementary material for: Perceived water-related risk factors of Buruli ulcer in two villages of south-central Côte d’Ivoire
Source: PLoS Negl Trop Dis. 2022 Dec 14;16(12):e0010927. doi: 10.1371/journal.pntd.0010927 (PMC9750022; doi:10.1371/journal.pntd.0010927)
Supplement: Attachment — Submitted filename: 20221015_Buruli_PBP_V5_fin.pdf [file pntd.0010927.s001.pdf]

Review of manuscript entitled "Perceived water-related risk factors of Buruli ulcer in two villages of south-central Côte d'Ivoire" (reference no. PNTD-D-22-00924)

Dear Paul J. Converse, dear Ana Nascimento

We were delighted to receive your e-mail dated 13 September 2022 providing us with the opportunity to revise the aforementioned manuscript. We are grateful for the encouraging feedback and constructive comments and suggestions from three external reviewers. We revised our piece in light of the reviewers' comments and tried our level best to address the concerns raised. In order to readily assist you in tracking our changes, they have been highlighted using a yellow marker. We also enclose a "clean" version of the revised manuscript and send you the point-by-point response below.

We very much hope that our revised manuscript is now suitable for publication in *PLoS Neglected Tropical Diseases*, and hence, we look forward to your further disposition.

Yours sincerely,
Andrea Leuenberger on behalf of all authors

Dear Dr. Leuenberger,
Your manuscript has been reviewed by three experts in the field. As you can see there is a wide range of issues to be addressed and suggestions of where the manuscript should be revised. Please address all the criticisms and provide more details about the subjects lesions and also discuss the obstacles to more effective implementation of health education measures.

Reviewer's Responses to Questions

Key Review Criteria Required for Acceptance?
As you describe the new analyses required for acceptance, please consider the following:

Methods
-Are the objectives of the study clearly articulated with a clear testable hypothesis stated?
-Is the study design appropriate to address the stated objectives?
-Is the population clearly described and appropriate for the hypothesis being tested?
-Is the sample size sufficient to ensure adequate power to address the hypothesis being tested?
-Were correct statistical analysis used to support conclusions?
-Are there concerns about ethical or regulatory requirements being met?

Reviewer #1: This is a qualitative study aiming to understand perceptions about Buruli ulcer transmission.

It did not aim to identify any association between behaviour and disease, which would need a much larger sample and a control group.

Response: We appreciate the feedback from Reviewer #1, including this concise summary.

Reviewer #2: The report of this study is part of a major study being undertaken in the study area. This is well described in the manuscript and the objectives for this qualitative study and how it relates to the larger study clearly outlined. The Prefecture and communities selected for the study are reported nationally as BU endemic areas making them appropriate for such a study. Prevalence of confirmed Buruli ulcer in communities is generally low, therefore, although the sample size is small, it is appropriate for the study being undertake, being qualitative. The information being solicited for could only be give by this sample size and therefore only percentages of demography was performed as a statistic. Lesion types were however not described in the sample as well as exact parts of the body where the lesions were located (extremities). This could be shown in a table.

Response: We are grateful to Reviewer #2 for the positive appraisal of our study and the constructive feedback. In line with this specific suggestion, we now provide further details about the wounds, as follows: "All except one Buruli ulcer lesion were located on the extremities (10 on lower legs and one on forearm). The remaining wound was located on the chest of a patient. All lesions had ulcerated, requiring treatment at the Buruli ulcer pavillon in Taabo. After the wound management in Taabo, patients returned to their villages and were followed-up by community health workers. All wounds were healed at the time of the follow-up interviews" (**see revised manuscript, lines 212-217**).

Regulatory and ethical requirements were met as the study was within the larger project for which ethical clearance has been granted. The study however failed to mention treatment of the patients and the confirmation laboratories used.

Response: We now specify that patients received standard treatment, namely rifampicin at 10 mg/kg body weight and clarithromycin at 7.5 mg/kg body weight for eight weeks (**see revised manuscript, lines 129-130**). Furthermore, we now provide details of the national reference laboratory of the Institut Pasteur in Abidjan, where samples of the patients were analyzed (**see revised manuscript, lines 141-143**).

Reviewer #3: Here is a qualitative study that aims to investigate the perceived water-related risk factors of Buruli ulcer in south-central Côte d'Ivoire
Even if it is a qualitative study, the number of subjects included seems to us to be very unrepresentative of the south-central Ivory Coast. (12 subjects included in 2 villages) How did you choose these two villages? How many villages does Taabo district have?
What is the WASH infrastructure coverage?

Response: In view of Reviewer #3's critique, we now provide further specific details about the study area, as follows: "Ahondo and Sahoua make up the Ahondo Health area, which has been the focal area of the framing research project. Since 2019, standardized case

report forms are utilized for tracking the wounds and active case detection was performed in both villages through household surveys in 2019 (May-July) and early 2022 (Toppino et al., 2022a, accepted). Notably, both villages, along with another 11 villages, form the Taabo health and demographic surveillance system (HDSS) (Koné et al., 2014)" (**see revised manuscript, lines 144-150**).

Is there a reason why the authors did not organize focus group discussions in order to compensate for the relatively small number of subjects included? I therefore suggest reviewing the title and clarifying the perceived water-related risk factors of Buruli ulcer in the localities investigated.

Response: We thank Reviewer #3 for highlighting the importance of focus group discussions (FGDs). We consider this study as primary inquiry with the patients (or their relatives). To be able to triangulate the perception of individual cases, we have complemented the communities' perspective by interviewing community health workers. Indeed, conducting FGDs with community members would have been insightful and should be considered for future investigations to better characterize the perception of the communities. Based on our initial insight, FGDs shall be used to scale up the study investigate further using a community-based approach. We revised the title as follows: "Perceived water-related risk factors of Buruli ulcer in two villages of south-central Côte d'Ivoire" (**see revised manuscript, lines 2-3**).

Results
-Does the analysis presented match the analysis plan?
-Are the results clearly and completely presented?
-Are the figures (Tables, Images) of sufficient quality for clarity?

Reviewer #1: The results are clearly stated.

Response: Thanks for this encouraging statement.

Reviewer #2: The objective of the study was to elicit for perceived risks of BU infection in the study communities and it targeted only reconfirmed patients. This could be described as preliminary to further investigation into the transmission of the Mycobacterium ulcerans and its reservoirs. The selection and interviews of the patients produced the required information. In the analysis, a better description of the exact positions and sizes of the lesions or nodules could have supported the exact risks to infection (socioeconomic activities, recreational activities, etc). Photos of the lesions should be presented to support selection of cases (if the ethical clearance allows).

Response: We are grateful for the constructive feedback regarding the lesions of the participants. Please note that photos of lesions have been presented elsewhere, within the frame of the lager research project. This is explained and we provide cross-reference to the key papers (**see revised manuscript, Toppino et al., 2022a, Toppino et al., 2022b**).

In the section from line 216 (Spatial Distribution of BU cases), the low

number of cases in this study provides a challenge to determining spatial distribution of cases. The title of this section should be edited to reflect the discussion of household cases as in the text.

Response: We agree, and hence, the heading of this section has been reworded as follows: "Geographic location and spatial distribution of Buruli ulcer cases' households" (**see revised manuscript, line 228**).

Healthcare workers were interviewed but little is reported of the results of those interviews and what it meant to the study or the information gathered from them.

Response: We appreciate this observation. Health workers were indeed interviewed, in an effort to triangulate the perspectives of affected people. Overall, the interviews with the health workers were in line with the statements put forward by community members. To enhance clarity, we have emphasized this point throughout the Results section as follows:

- "Furthermore, almost half of the participants emphasized the irremediable or even fatal consequences of an infection, as illustrated in the following quote from a health care worker: "In the [patient's] yard there, almost everyone had Buruli ulcer. It was this kind of wound that killed his mama. Those wounds killed his little brother. The wounds killed his big sister's child. [Name] had Buruli ulcer, there's [name] who had Buruli ulcer, there's the son that had Buruli ulcer. Huh! She even had Buruli ulcer. Almost everyone in their yard." (CHW_2)" (**see revised manuscript, lines 234-242**).
- "As unimproved water sources, few participants dug their own wells in close proximity to their houses (Fig 2A). Water holes visited were usually not protected. Natural occurring surface water bodies were also reported as important sources, both by cases and health care workers. For all participants, the Bandama River was a key water source throughout the year, but especially during the dry season (Fig 2B, Fig 2D). Further, nearly all participants use seasonal low-lands ("bas fonds") and ponds ("marigots"), which are – when filled with water – closer to their houses than the river. Health care workers suspected contact with these natural water sources (river, low-lands, and ponds) to increase the risk of infection with *M. ulcerans*" (**see revised manuscript, lines 256-264**).
- "Different factors on the communal or societal level influence the nature of water exposure of residents. For example, depending on the availability, functionality, and reliability of water infrastructure (taps and pumps), people may depend more on improved than surface water sources. Some health care workers indicated that they believe that, in particular, the availability and reliability of running water in the community is a key factor influencing the incidence of Buruli ulcer. Additionally, water contact patterns were governed by gender division of labor. When asked about their patterns of contacting natural water sources, males mentioned primarily agricultural chores. Females, on the other hand, reported mostly specific household activities and specific care work for children" (**see revised manuscript, lines 331-340**).
- "Others frequent the river because they are used to it, as explained by a health care worker: "I can say that it is a question of habit. The parents are used to drink the water of the river. We can only tell them not to drink the water from the Bandama or the ponds. That's it! Otherwise, we can say in Sahoua there are many pumps, but as it is a habit for people to drink other waters […] we cannot change it." (CHW_1)" (**see revised manuscript, lines 353-359**).

In the discussion (lines 368 – 371), the authors estimated the prevalence as a percentage for the communities based on the sample size. The description there is problematic as they failed to use the actual cases over a specific period of time for these estimates. Moreover, the authors could have easily verified the claim that Buruli ulcer has been fatal to some family members of the participants. The HDSS system in Taabo could provide this information.

Response: Indeed, the prevalence is a rough estimation, and hence, does not consider influencing factors such as socioeconomic status, family history, or seasonality. It would be interesting to compare the reported fatality from our study with data from the verbal autopsies from the Taabo HDSS. However, as we do not have access to the Taabo HDSS data, this point is added as a study limitation, as follows: "Second, our study focused on Buruli ulcer cases from two endemic villages. The sample size with only 12 cases was limited and no control villages were included. Of note, data saturation was attained by the 12 interviews and cases' perspectives were complemented by interviews with health care workers. It would have been interesting to triangulate our findings with secondary data such as verbal autopsies of the Taabo HDSS" (**see revised manuscript, lines 461-466**).

Reviewer #3: The results of the study are certainly interesting but do not really bring new data.
In our opinion, the results must be better studied in order to bring out the deep mechanisms underlying the choices of the populations and to see with them the levers on which we can base ourselves to change the situation within the framework of a "problem solving" approach.
Can the authors give us an overview of the number of BU cases in the districts. and in the villages included over time?.

Response: We appreciate this feedback and agree that our study confirms observations made elsewhere. We view our study as a pilot. More in-depth investigations are warranted that will help to elucidate the distribution of Buruli ulcer, including underlying risk factors. Based on the data from the current study, we cannot provide longitudinal data about the prevalence of Buruli ulcer in the district. For more details, we kindly refer to publication from the framing research project about the burden of wounds in the area, showcasing the findings from a household-wound-survey (Toppino et al., 2022b; accepted for publication).

Conclusions
-Are the conclusions supported by the data presented?
-Are the limitations of analysis clearly described?
-Do the authors discuss how these data can be helpful to advance our understanding of the topic under study?
-Is public health relevance addressed?

Reviewer #1: The main message is stated as: "this study specifically raises the voice of affected populations, calling for increased awareness and improved knowledge to combat this neglected tropical skin disease and

reduce its health, social, and economic consequences." I disagree with this - I think that the study shows that knowledge and awareness about BU are quite well developed in these villages, where various projects have been operating for at least 20 years (I have been there myself several times!). The key issue is that people are not motivated to put this knowledge into practice. This is the fundamental obstacle faced by all programs of health education or health promotion. Reorienting the Discussion to this problem would make the paper more insightful.

Response: We thank Reviewer #1 for this candid feedback. Having observed many patients ourselves, we assume that overcoming stigmatization of wounds in general is a key aspect for early treatment. This issue is now highlighted more clearly in the Discussion: “Including the community perspective to further investigate the complex interplay of environmental, cultural, and behavioral risk factors is therefore inevitable. Especially culturally appropriate and effective interventions, which can be easily adopted by affected communities, should be designed and implemented. Indeed, community engagement is key to actively prevent Buruli ulcer and allowing community members to contribute to the early identification of cases. Besides community-based interventions, the community’s perspective is important to inform large-scale environmental sampling” (**see revised manuscript, lines 437-444**). Additionally, we have reformulated the sentence mentioned above, to read: “While official Buruli ulcer case numbers might be underreported, this study specifically raises the voice of affected populations, calling for increased awareness and community engagement to combat this neglected tropical skin disease and reduce its health, social, and economic consequences” (**see revised manuscript, lines 484-488**).

Reviewer #2: The conclusions presented in the manuscript reflect the information sought by the study and the objectives. Results from this study confirms the need to map environmental contacts communities when undertaking ecological and transmission studies of Mycobacterium ulcerans. The authors also used information gathered in the interviews to suggest the need for tailormade designs for interventions in Buruli ulcer endemic communities.

Response: We are grateful for these generous comments.

Reviewer #3: No specific comments

Response: We take it that Reviewer #3 is content with our Conclusions.

Editorial and Data Presentation Modifications?
Use this section for editorial suggestions as well as relatively minor modifications of existing data that would enhance clarity. If the only modifications needed are minor and/or editorial, you may wish to recommend “Minor Revision” or “Accept”.

Reviewer #1: Revision of the Discussion would be helpful.

Response: In line with Reviewer #1’s comment, we have amended the Discussion, emphazing the need of community engagement. Additionally, the need for community engagement is also stressed in the Conclusion, as explained before (**see revised**

manuscript, lines 484-488).

Reviewer #2: To improve the paper, the authors:
1. Improve on their case selection criteria by describing the lesions and adding photos.
2. Edit title on line 216 to reflect content of paragraphs
3. Adjust estimates of prevalence with actual case numbers and not only samples
4. Add information gathered from health workers
5. Verify fatality of Buruli ulcer in the DHSS.

Response: We have addressed these issues, point-by-point:

1. We have integrated more details about the location of the wounds and characteristic of the lesions. For further details (including photographs), we kindly refer the interested reader to a paper from the framing research project, which has been accepted for publication in PLoS Neglected Tropical Diseases (Toppino et al., 2022b).
2. This sub-title is amended to read: "Geographic location and spatial distribution of Buruli ulcer cases' households" (**see revised manuscript, lines 228**).
3. To address this point, we have elaborated our interpretations and provide cross-reference to a key publication: "In line with findings from household wound surveys from the framing research project (Toppino et al., 2022a), our qualitative inquiry offers indication that Buruli ulcer remains endemic in the Taabo HDSS (Koné et al., 2014)" (**see revised manuscript, lines 389-392**).
4. Information obtained from health care workers has been emphasized throughout the Results section (**see revised manuscript, lines 258ff, 263f and 334ff**). Additionally, we have indicated two quotes from health care workers (**see revised manuscript, lines 235ff and 353ff**).
5. As explained above, we greatly appreciate this input and have noted it for future studies.

Reviewer #3: (No Response)

Summary and General Comments
Use this section to provide overall comments, discuss strengths/weaknesses of the study, novelty, significance, general execution and scholarship. You may also include additional comments for the author, including concerns about dual publication, research ethics, or publication ethics. If requesting major revision, please articulate the new experiments that are needed.

Reviewer #1: Revision of the Discussion would be helpful.

Response: We carefully revised the Discussion and Conclusoins and highlighted the importance of community engagement more forcefully (**see revised manuscript, lines 439-445 and 484-488)**.

Reviewer #2: This paper reports on a very pressing public health problem and has added valuable information on the importance of environmental

mapping in addressing the transmission issues of Buruli ulcer. Buruli ulcer remains a disease of public health importance and the selection of the study communities is appropriate to the study. The authors also clarified that this study was part of a larger study. A main weakness of this paper is the number of cases that were interviewed, but being largely qualitative, information gathered from the patients is valuable and its use appropriate to the objectives of the study.

Response: Thanks for these generous comments. We agree that the number of cases interviewed (*n* = 12) is quite small. However, data saturation was reached by interviewing 12 patients, indicating that we conducted a sufficient amount of interviews. Additionally, the perspectives of cases and health care workers were mostly congruent and thus, underlining data saturation. We have clarified this in the limitation sections of our manuscript (**see revised manuscript, lines 461-466**).

Reviewer #3: In the current state of the manuscript, it seems to us that the manuscript requires a major revision. The methodology and results section deserve to be better explained and more detailed.
Failing this, the authors must review the title and the objectives of the study in order to report a preliminary study to be deepened by subsequent investigations.

Response: In line with points offered by Reviewer #3, we provide additional details in the Methods and Results sections. In our view, it is important to reflect the communities' perspectives, and hence, we offered several key areas that warrant further investigations, such as gender-specific risk factors (**see revised manuscript lines 422ff**), seasonality of risk factors and disease incidence (**see revised manuscript line 379ff**) or large-scale environmental sampling (**see revised manuscript lines 379ff**). It follows that our qualitative insights might guide future studies to unravel this mysterious disease to better tailor preventive and curative interventions to fight Buruli ulcer at the community level.

References

Koné S, Baikoro N, N'Guessan Y, Jäger FN, Silué KD, Fürst T, et al. Health and demographic surveillance system profile: the Taabo Health and Demographic Surveillance System, Côte d'Ivoire. International Journal of Epidemiology. 2015;44(1):87-97.

Toppino S, Koffi DY, Kone VB, N'Krumah RTAS, Coulibaly ID, Tobian F, et al. Community-based wound management in a rural setting of Côte d'Ivoire. PLoS Negl Trop Dis. 2022a (accepted for publication).

Toppino S, N'Krumah RTAS, Kone VB, Koffi DY, Coulibaly ID, Tobian F, et al. Skin wounds in a rural setting of Côte d'Ivoire: population-based assessment of the burden and clinical epidemiology. PLoS Negl Trop Dis. 2022b (accepted for publication).
